# Gelatin nanoparticles enable water dispersibility and potentialize the antimicrobial activity of Buriti (*Mauritia flexuosa*) oil

**DOI:** 10.1186/s12896-020-00649-4

**Published:** 2020-10-16

**Authors:** Gabrielle Mahara Martins Azevedo Castro, Thais Souza Passos, Sara Sayonara da Cruz Nascimento, Isaiane Medeiros, Nathália Kelly Araújo, Bruna Leal Lima Maciel, Carlos Eduardo Padilha, Adriana Margarida Zanbotto Ramalho, Francisco Canidé Sousa Júnior, Cristiane Fernandes de Assis

**Affiliations:** 1 Nutrition Postgraduate Program, Center for Health Sciences, Federal University of Rio Grande do Norte, Natal, RN Brazil; 2 Department of Nutrition, Center for Health Sciences, Federal University of Rio Grande do Norte, Natal, RN Brazil; 3 Department of Pharmacy, Center for Health Sciences, Federal University of Rio Grande do Norte, Natal, RN 59078-970 Brazil; 4 Federal Institute of Education, Science and Technology of Rio Grande do Norte, Pau dos Ferros, RN Brazil; 5 Department of Chemical Engineering, Federal University of Rio Grande do Norte, Natal, RN Brazil; 6 Animal Nutrition Postgraduate Program, Academic Unit of Agricultural Sciences, Jundiaí Agricultural School, Macaíba, RN Brazil

**Keywords:** Vegetable oils, Fatty acids, Phenolic compounds, Emulsification O/W

## Abstract

**Background:**

Buriti oil presents numerous health benefits, but due to its lipophilic nature and high oxidation, it is impossible to incorporate it into aqueous food matrices. Thus, the present study evaluated whether powder nanoparticles based on porcine gelatin (OPG) and in combination with sodium alginate (OAG) containing buriti oil obtained by O/W emulsification followed by freeze-drying enabled water dispersibility and preserved or increased the antimicrobial activity of the oil.

**Results:**

OPG presented spherical shape, smooth surface, smaller particle size and polydispersity index [51.0 (6.07) nm and 0.40 (0.05)], and better chemical interaction between the nonpolar amino acids and the hydrophobic oil chain. OPG also presented a higher dispersibility percentage [85.62% (7.82)] than OAG [50.19% (7.24)] (*p* < 0.05), and significantly increased the antimicrobial activity of the oil by 59, 62, and 43% for *Pseudomonas aeruginosa*, *Klebsiella pneumonia,* and *Staphylococcus aureus*, respectively.

**Conclusions:**

Thus, nanoencapsulation in gelatin is a promising strategy to increase the potential to use buriti oil in foods.

## Background

The food industry has a growing demand for vegetable oils from a wide range of natural sources, especially the development of formulations of products and other applications [1]. Nonconventional vegetable oils have been considerably investigated due to their constituents’ chemical properties, which increase the potential for functional applications such as food, cosmetics, and pharmaceuticals [2].

Buritizeiro (*Mauritia flexuosa*) is a native palm of the Amazon Region that can be found in the Cerrado and seasonally, in Northeast and Central-South Brazil [3]. This palm offers buriti, a nutritious fruit known for its orange-yellow flesh, from which an oil with a high and predominant concentration of carotenoids, mainly *β*-carotene, *α*-carotene, and zeaxanthin can be extracted [4].

This oil presents antioxidant characteristics and high oleic fatty acid content [5]. The bioactive compounds present in buriti oil promote a significant antioxidant capacity, and their consumption may reduce the risks of developing noncommunicable chronic diseases (NCDs). These properties gave a market appeal for the use of buriti in industrialized food [6]. Besides, naturally occurring antimicrobial agents are extensively studied for their potential application in the food industry [7]. Studies have shown dewormer and antimicrobial effects of buriti oil [8].

However, the insolubility in water, undesirable flavors, and instability of some bioactive compounds due to the exposure of factors related to food processing and storage, such as heat, light, and oxygen, make it challenging to apply vegetable oils in aqueous food matrices [9]. The encapsulation can be used to protect oils against lipid oxidation, and control the release of bioactive substances, such as carotenoids and phenolic compounds, being of great interest [10]. Therefore, encapsulates containing vegetable oils showed promising results regarding the acceptability of sensorial aspects and the stability of fatty acids and phenolic compounds [11].

In this context, nanoencapsulation promotes the increase in the water solubility of lipophilic substances, preserves or enhances the biological properties, and increases compounds bioavailability. These positive effects make the development of new ingredients and products feasible [12, 13]. The emulsification technique is an effective physical-chemical method to promote the encapsulation of lipophilic substances and is increasingly used in the food industry [14]. Besides, the conversion of a liquid emulsion to oil in powder using drying techniques such as spray-drying or freeze-drying can also allow extensive use as an alternative ingredient and facilitate its use in the food industry [15].

Alginate is an anionic biopolymer of high molecular weight composed of linear chains of β-D-mannuronic acid joined by type bonds (1 → 4) and residues of its epimer, α-L-guluronic acid. They form hydrogels, films, micro, and nanoparticles in the presence of ions (Ca^++^), capable of encapsulating numerous substances of a variable nature [16].

Gelatin is a protein resulting from the controlled hydrolysis of collagen from different animal sources (porcine, bovine, and fish), with applications in the food, pharmaceutical, and cosmetic industries. It presents amphoteric behavior in solution, which allows it to behave like acid and base depending on the solution’s pH. Research shows that gelatin’s ability to act as an emulsifier in oil-in-water emulsions, promotes an increase in the physicochemical stability of polyunsaturated fatty acids [17, 18]. This is a promising application for the food sector [19]. Alginate, being an anionic polymer, can interact with proteins in solutions with pH values below the protein’s isoelectric point, contributing to improving the stability of oil-in-water emulsions [20]. Based on this, the present study aimed to evaluate the isolated and conjugated effects of porcine gelatin and sodium alginate on obtaining powder particles containing buriti oil, evaluating the effect of encapsulation on the water dispersibility and antimicrobial activity of buriti oil.

## Methods

### Chemicals

The buriti oil obtained from the pulp and peel of the species *Mauritia flexuosa* was donated by the company Plantus® in the Rio Grande do Norte - Brazil. Porcine gelatin (Type A), sodium alginate, and Tween 20 were purchased from Sigma-Aldrich®.

### Buriti oil characterization

#### Determination of the fatty acid profile by gas chromatography (GC)

For the analysis of the fatty acids of buriti oil by GC, methylation was performed previously, according to the method described by Hartman and Lago [21] to obtain the fatty acid methyl esters (FAMEs).

GC (Thermo Scientific - CG / FID - FOCUS) with a flame ionization detector (FID) and Supelco SP2560 capillary column (100 mm × 0.25 mm × 0.2 μm) identified and quantified the FAMES using nitrogen gas as a carrier gas (2.5 ml.min^− 1^). The programming of the column temperature rise was 40 °C for 3 min. It was heated to 180 °C for 5 min at a rate of 10 °C.min^− 1^ and heated again at 220 °C for 3 min at a rate of 10 °C/min, and finally, the temperature reached 240 °C maintained for 25 min at a rate of 20 °C/min. The injector and detector temperatures were 230 °C and 270 °C, respectively.

The injected sample volume was 1 μL with a 10:1 split ratio. The peaks were integrated and compared to the fatty acid standards (SupelcoTM37 component FAME MIX).

#### Determination of phenolic compounds

The hydrophilic fraction (HF) of buriti oil was obtained, according to Espín et al. [22], with modifications. A ratio of buriti oil and methanol of 1:1 (w/v) was vortexed (Phoeniz, model AP59) for 1 minute and then centrifuged (7560 x g/15 min) (FANEM, Excelsa 4, model 280R). HF was collected until the exhaustion of the supernatant color. Then, HF was concentrated in a rotary evaporator (Buchi, R-100) at 27 °C and dried using nitrogen flow.

Subsequently, HF was solubilized in acetonitrile (0.1 mg.mL^− 1^), and the solution was filtered through a 0.22 μm syringe filter before analysis by high-performance liquid chromatography (HPLC) on an Acella chromatograph (Thermo Scientific) coupled to the detector by diode array (PDA). Chromatography was performed according to Kim et al. [23] with modifications. A reverse-phase column Shim-Pack CLC-ODS (M) C18 (25 cm × 4.6 mm) consisting of silica modified by octadecyl groups, with pores of 5.0 μm in diameter, maintained at 40 °C was used.

The mobile phase composition consisted of water (Phase A) and acetonitrile (Phase B), containing 1% acetic acid, with a flow rate of 1.0 mL/minute. Elution was performed using a linear gradient of 0 to 30% B in 10 min, 30 to 70% B in 5 min, 70 to 100% in 10 min, and 100% B maintained for 5 min. Calibration curves were performed with the standards (Sigma®) of gallic acid (280 nm), catechin (280 nm), eugenol (280 nm), vanillic acid (280 nm), syringic acid (280 nm), ellagic acid (256 nm), vanillin (280 nm) and quercetin (255 nm). The results were expressed as the mean and standard deviation per μg.g^− 1^ of buriti oil.

### Encapsulation of the Buriti oil

The particles loaded with buriti oil were obtained by the O/W emulsification technique, followed by dispersion of solution containing encapsulating agent in the emulsion obtained, based on Medeiros et al. [24] with modifications.

The encapsulating agents used were porcine gelatin, alginate, and a combination of both and Tween 20 as a surfactant. The formulations were (1) buriti oil and porcine gelatin (OPG) and (2) buriti oil, alginate, and gelatin (OAG).

The oil phase was 10 mL of buriti oil for both groups. The aqueous phases (FA) were formulated as follows: FA 1 (90 mL) consisted of 1.5% (w/v) Tween 20 solubilized in distilled water for all groups, and FA 2 (100 mL) contained 4% (w/v) of the encapsulating agent and 1.5% (w/v) of Tween 20 solubilized in distilled water. FA 2 of the encapsulating agent in OAG contained 3% (w/v) porcine gelatin and 1% (w/v) sodium alginate.

Sodium alginate was solubilized in distilled water under magnetic stirring for 24 h at 50 °C. Then, the solution was filtered on qualitative filter paper. The porcine gelatin was solubilized in distilled water under magnetic stirring at 40 °C, according to Medeiros et al. [24]. Finally, the solution containing sodium alginate and porcine gelatin (OAG) was obtained from alginate solubilization, with subsequent homogenization of the porcine gelatin solution under magnetic stirring for 1 h at 40 °C. Finally, the pH of the solution was adjusted to 5.5 using HCl PA.

The emulsion was obtained by ultradispersion of FA 1 with the oil phase (Ultra-Turrax, IKA® T18 basic) at 17.000 rpm/10 min. Subsequently, FA 2 was dispersed in the obtained emulsion using the same conditions as described above. Finally, according to Medeiros et al. [24], the formulations obtained in triplicate were freeze-dried (LioTop L101) at − 57 °C and pressure of 43 μHg for subsequent particle characterization, dispersion assay, and potential antimicrobial evaluation.

### Characterization of particles

#### Scanning electron microscopy (SEM)

Powdered particles were dispersed in acetone and dripped on silicon chips fixed in stubs to determine the morphology. The analysis was performed at different magnifications, using a high vacuum, 2–3 kV voltage, and without metallization, under the MEV-FEG ZEISS microscope (AURIGA).

#### Laser diffraction

The particle size determination was based on Medeiros et al. [24]. Ten milligram of each encapsulated powder was dispersed in acetone under magnetic stirring at room temperature for 2 min. Subsequently, 2 mL of formaldehyde was added, and the dispersions were stirred for 30 min to promote particle deagglomeration. The dispersions were filtered, and the particles retained on the qualitative filter paper were collected and redispersed in 4 mL of acetone.

All variables of this process were standardized through tests. The dispersions were placed in glass cuvettes and read at 5 runs/1 min to measure the mean diameter and polydispersion index in the NanoBrook ZetaPlus Zeta Potential Analyzer, Brookhaven Instruments - ZetaPALS Particle Sizing Software. The whole procedure was performed in triplicate.

#### Zeta potential

For this measure, 10 mg of each powdered encapsulate was dispersed in 4 mL of distilled water and placed in specific cuvettes. Ten runs in 01 min were performed, and the NanoBrook ZetaPlus Zeta Potential Analyzer, Brookhaven Instruments - PALS Zeta Potential Analyzer software was used.

#### Fourier transform infrared spectroscopy (FTIR)

Buriti oil, sodium alginate, porcine gelatin, Tween 20, OPG, and OAG were homogenized in potassium bromide (KBr), macerated, and pressed into pellets.

Subsequently, they were recorded in the transmittance and medium infrared region from 400 to 4000 cm^− 1^. The Shimadzu spectrometer, model FTIR-8400S, IRAFFINITY-1 series, IRSOLUTION software, version 1.60, with 32 scan numbers and 4 cm^− 1^ resolution was used.

#### X-ray diffraction

The encapsulating agents, OPG and OAG were analyzed using a high-resolution X-ray diffractometer (SHIMADZU, model XRD 7000) with a Seifert ID3000 generator to evaluate the dominant phase in the materials (crystalline or amorphous). For this analysis, the encapsulating agents and powdered encapsulates were placed in a cylindrical sample holder and analyzed at a 2ɵ diffraction angle between 0 and 50°.

### Total Buriti oil determination

The lipid amount (buriti oil) present in formulations was determined, according to Calvo et al. [25], with modifications. One gram of each sample was placed in the Soxhlet extractor for 24 h, with approximately 200 mL of hexane. The total extracted oil was weighed and expressed in percentages according to the equation: Buriti oil (%) = buriti oil present in powder formulation (g) / total buriti oil used in the process (g) × 100. The procedures were performed in triplicate, and values were expressed as the mean and standard deviation.

### Water dispersion assay

The assay was performed according to Eastman and Moore [26] with modifications. 200 mg and 20 mg, respectively, of the encapsulated formulations and crude buriti oil were dispersed in 4 mL of distilled water in test tubes in an orbital shaker (QUIMIB® - Q816M20) for 48 h at 27 °C (± 2 °C) at 120 rpm.

After 48 h, the materials were centrifuged (CENTRIBIO) at 3000 x g for 5 min to promote the separation of lipid fractions, which were transferred to previously tared porcelain capsules. The capsules were placed in an oven at 105 °C for 5 h and weighed to determine the percentage (%) of no solubilized material, subtracted from the total content to obtain the amount of solubilized material. The procedure was performed in triplicate, and the results were expressed in percentages.

### Antimicrobial activity

The antimicrobial effects against *Klebsiella pneumonia* (ATCC10031), *Pseudomonas aeruginosa* (ATCC27853), and *Staphylococcus aureus* (ATCC6538) were evaluated only in the formulation that presented the best results for the characterization of the particles, total buriti oil determination, and dispersibility in water.

In a 96-well plate, 50 μL of the 10^5^ CFU.mL^− 1^ bacterial suspensions in Müeller Hinton broth (MH, Himedia, India) were added to the solution containing crude buriti oil (5 mg.mL^− 1^), OPG (5 m*s*g.mL^− 1^ of buriti oil present in OPG), vancomycin (0.4 mg.mL^− 1^) and gentamycin (0.3 mg.mL^− 1^) incubated at 35 °C under shaking at 200 rpm. The optical density at 595 nm was determined in a microplate reader (Epoch Biotek, Winooski, USA) at times 0 and 24 h. Wells with medium and saline solution were used as medium sterility controls (negative growth control).

The inhibitory capacity (%) against the strains tested was calculated concerning the growth control group (untreated, considering 100% growth). Crude buriti oil was used as a control and solubilized in a solution containing Tween 80 and distilled water in the proportion of 1:1 v/v. Porcine gelatin and the solution of Tween 80 and distilled water (1: 1 v/v) were also used as control at the same concentration present in OPG.

### Statistical analysis

The results were expressed as the mean and standard deviation. The results were expressed as the mean and standard deviation. ANOVA and Tukey post hoc tests were used to compare the results obtained for the buriti oil (%) presented in OPG and OAG, and the water dispersion assay. Student’s t-test was used to evaluate the average growth inhibition percentages obtained for buriti oil and OPG formulation against the tested microorganisms. Statistical analysis was performed using GraphPad Prism software version 5.0, considering *p* < 0.05 the significance level.

## Results and discussion

### Fatty acids profile

Gas chromatography, considered an essential technique for research and development, or only quality control, allows the complete identification and quantification of each type of fatty acid present in food products [27]. In the present study, the buriti oil presented a predominance of palmitic acid 25.48% (0.37) (Table 1), which was higher than the values found in the literature that varied between 17 and 19% [2, 28]. The linoleic fatty acid content (52.54%) (1.15) (Table 1) was even higher than that in studies with conventional crude and refined buriti oil [29]. Thus, the buriti oil evaluated presented the potential to be used as an ingredient in industrialized foods, since it had a high content of polyunsaturated fatty acids (52.79%) (1.15).

**Table 1:** Fatty acid profile of buriti oil (*M. flexuosa*) determined by Gas Chromatography of fatty acid methyl esters

Fatty acid	%
C14:0 (Myristic acid)	0.85 (0.03)
C16:0 (Palmitic acid)	25.48 (0.37)
C16:1 (Palmitoleic acid)	0.45 (0.01)
C17:0 (Heptadecanoic acid)	0.05 (0.03)
C18:0 (Stearic acid)	1.97 (0.87)
C18:1 (Elaidic acid)	1.56 (0.74)
C18:1n9c (Oleic acid)	17.03 (0.35)
C18:1n9t (Elaidic acid)	0.42 (0.19)
C18:2n6c (Linoleic acid)	52.54 (1.15)
C18:2n6t (Linolelaidic acid)	0.24 (0.00)
C20:1 (Eicosanoic acid)	0.14 (0.05)
C21:0 (henicosanoic acid)	0.08 (0.03)
C22:0 (Behenic acid)	0.07 (0.03)
Saturated Fatty Acids	28.5 (0.41)
Monounsaturated Fatty Acids	19.46 (0.20)
Polyunsaturated Fatty Acids	52.79 (1.15)

Mean and standard deviation (SD), *n* = 2

The oleic acid (C18:1n9c) percentage was 17.03% (0.35), and the absence of linolenic acid (ω-3) was observed. This distribution was different from that found in other studies, which obtained an average of 2% linolenic acid and a prevalence of more than 70% oleic acid, which classifies buriti oil as monounsaturated, even compared to olive oil [29, 30]. These differences may be related to the source of the raw material and the part of the fruit used to obtain the oil (peel and pulp) [31, 32].

The trans fatty acids elaidic (C18:1n9t) and linolelaidic acid (C18:2n6t), commonly found in processed foods and in fats that undergo hydrogenation [33] were found in the buriti oil analyzed at insignificant levels when compared to the parameters of the Codex Alimentarius [34].

### Content of phenolic compounds present in Buriti oil

The primary phenolic compound present in the buriti oil evaluated was quercetin (Table 2), which presents potent antioxidant power. Its properties are related to cardioprotective and gastroprotective effects, antihypertensive, antidiabetogenic, and immunomodulatory effects, protecting the body against cancer, inflammation, infections, and allergic processes [35].

**Table 2:** Profile of phenolic compounds present in the hydrophilic fraction of buriti (*M. flexuosa*) oil obtained by HPLC using the commercial standards of gallic acid, eugenol, catechin, vanillic acid, syringic acid, ellagic acid, vanillin, and quercetin

Phenolic compound	Mean (μg.g^**−1**^) (SD)
Galic acid	ND^a^
Catechin	0.33 (0.02)
Vanillic acid	3.49 (0.04)
Siringic acid	0.31 (0.02)
Ellagic acid	1.11 (0.04)
Vanillin	0.70 (0.31)
Quercetin	20.53 (0.37)
Eugenol	17.60 (0.16)

^a^Not detected

The second phenolic predominant in the buriti oil was eugenol [17.60 (0.16) μg.g^− 1^], lower than the average found, for example, in clove (*Syzygium aromaticum*), mentioned in the literature as one of the primary sources of eugenol. This phenolic compound can be found in essential oils extracted from leaves and fruits of plants such as *Pimenta dioica* [36]. This phenolic has antioxidant, antimicrobial, antiviral, and cytotoxic properties [37].

Vanillin was also detected at a concentration of 0.70 (0.31) μg.g^− 1^. This compound is characterized as an essential flavoring, and its most abundant source is the plant *Vanilla planifolia*, which is widely used in the food industry [38].

Tannins, ellagic acid, and catechin, antioxidants, were also detected in the study. These substances present antimicrobial and cytotoxic effects in organism, contributing to the prevention of oxidative stress induced by free radicals [39].

Therefore, according to Leão et al., [7], these constituents present in vegetable oils, such as buriti oil evaluated in the present study, promote an antioxidant effect through various chemical mechanisms, including the elimination of free radicals through electron transfer or donation of hydrogen and chelation of transition metals.

### Particle characterization

In the present study, we sought to evaluate powder formulations obtained by drying the emulsions, as well as in the studies by Comunian et al. [40] and Moser et al. [41] to expand the potential for use in aqueous matrix foods.

**Fig. 1:**
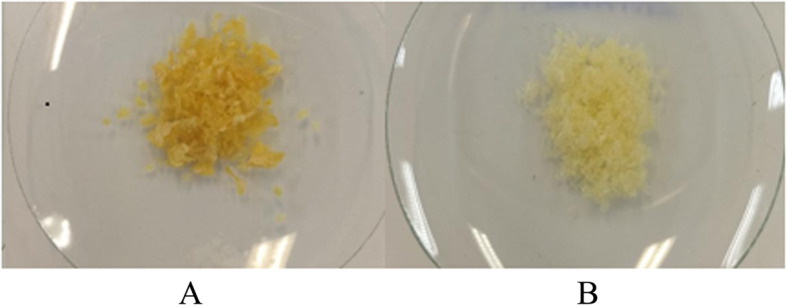
Powder particles loaded with buriti (*Mauritia flexuosa*) oil obtained by the O/W emulsification technique. **a** OAG: buriti oil encapsulated in the combination of sodium alginate and porcine gelatin; and (**b**) OPG: buriti oil encapsulated in porcine gelatin

The present study aimed to encapsulate buriti oil using porcine gelatin and combine it with sodium alginate (Fig. 1a and b). These materials were chosen because of their low cost and biodegradable, biocompatible, and nontoxic characteristics [42]. It is worth mentioning that there are no published data in the literature on the nanoencapsulation of buriti oil using carbohydrates and proteins as encapsulating agents.

The proportion of alginate combined with gelatin was established based on Soliman et al. [43]. They observed that high alginate concentrations promoted a reduction in the incorporation efficiency of essential vegetable oils. Thus, an increase in the space occupied by alginate causes a decrease in the free area, reducing the oil amount in the system.

#### Scanning electron microscopy (SEM)

**Fig. 2:**
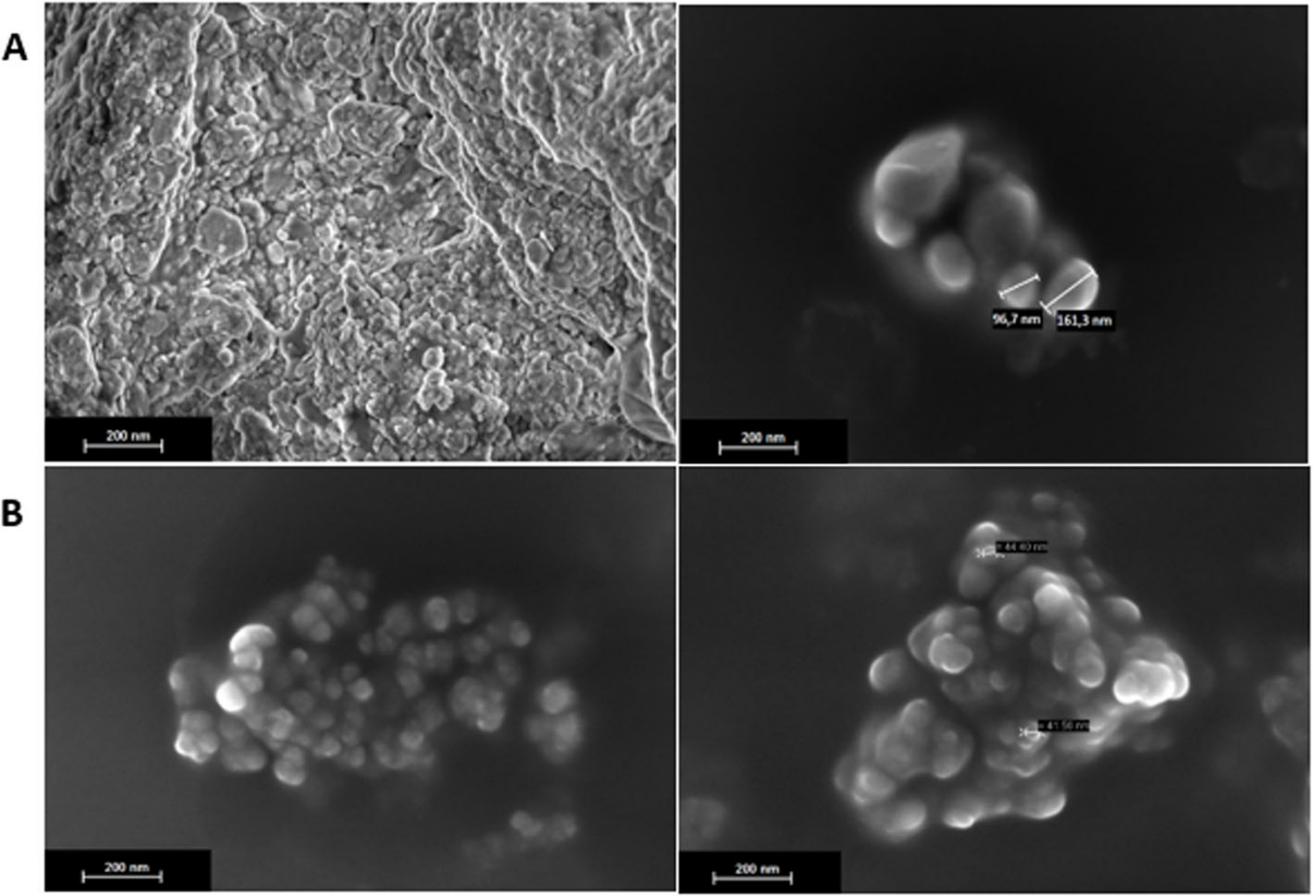
Micrographs of powder particles, obtained by the O/W emulsification technique, dispersed in acetone. **a** OAG: buriti oil, sodium alginate and porcine gelatin with a magnitude of 10.000 X and 70.000 X; (**b**) OPG: buriti oil, and porcine gelatin with a magnitude of 70.000 X and 80.000 X

SEM can analyze and characterize the surface morphological aspects of the particles, and their physical size and homogeneity [44, 45]. The OAG particles (Fig. 2a) were spherical and oval-shaped with smooth surfaces, without cracks or depressions. However, a large agglomeration and heterogeneity regarding the physical size distribution, with many particles on the micrometer scale and few on the nanometer scale was observed. Lemos et al. [46] microencapsulated buriti oil by complex coacervation, followed by freeze-drying using gelatin and sodium alginate as encapsulating agents in the same gelatin:alginate ratio used in the present study of 3:1 (w/w), and obtained multinucleated microparticles with irregular shapes and heterogeneous sizes.

OPG (Fig. 2b) particles were spherical with smooth surfaces and physical size on the nanometer scale (< 100 nm), with homogeneous size distribution and low agglomeration, compared to OAG. Thus, the buriti oil encapsulated by the O/W emulsification technique was protected in both formulations (OPG and OAG). However, porcine gelatin (OPG) was the encapsulating agent that provided a smaller and homogeneous size distribution.

#### Laser diffraction

Particle size is an essential parameter for defining the application of encapsulates. Sagiri et al. [47] emphasized that the distribution of particle size is of great importance when the intention is to incorporate ingredients in foods. Large particles with wide range size distribution can affect the texture attributes of the final product and the bioavailability of the incorporated bioactive compounds [48, 49].

**Fig. 3:**
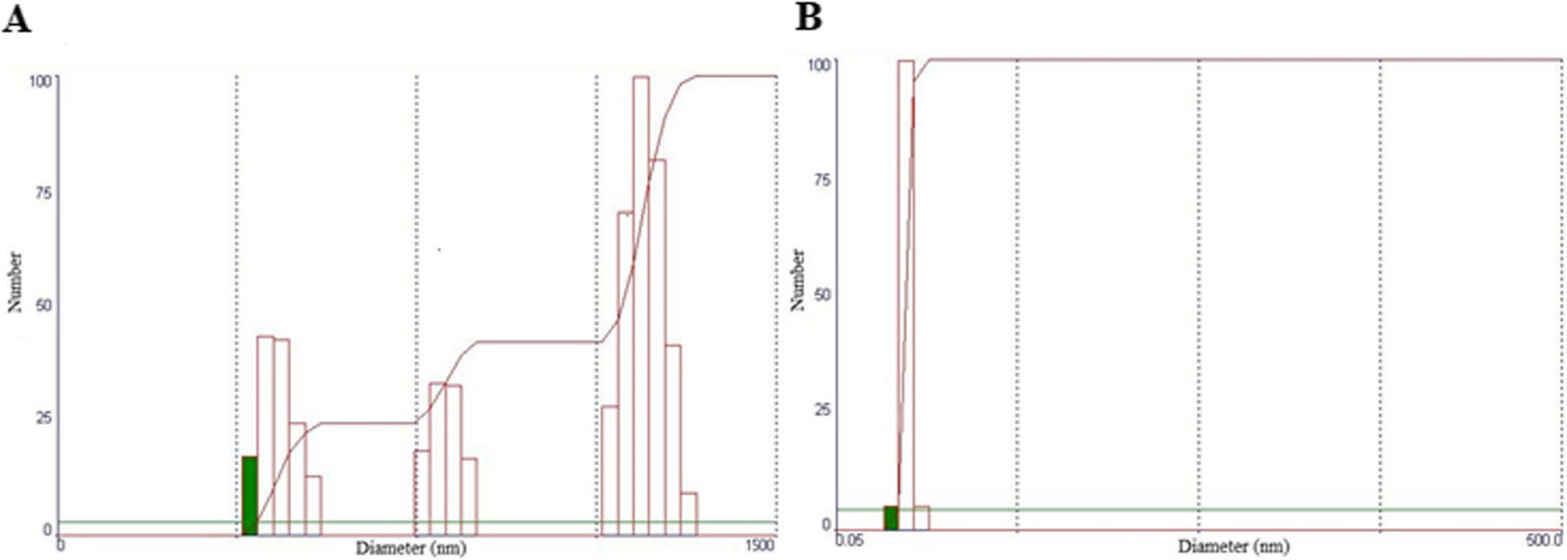
Particle size distribution by laser diffraction of encapsulated powder particles obtained by the O/W emulsification technique, which were cross-linked and redispersed in acetone for measurement. **a** OAG: buriti oil, sodium alginate, and porcine gelatin; (**b**) OPG: buriti oil and porcine gelatin

The particle size (Fig. 3) and polydispersity index results obtained for the formulations produced in the present study were 400 (7.0) nm and 0.55 (0.12) for smaller particles and 1443.87 (87.83) nm and 0.60 (0.09) for larger particles for the OAG formulation. Additionally, 51.00 (6.07) nm and 0.40 (0.05) for the OPG nanoformulation. Thus, OPG (Fig. 3b) presented unimodal size distribution, and OAG (Fig. 3a) showed a trimodal size distribution.

Based on this, the encapsulation of buriti oil with porcine gelatin promoted the nanoparticles (< 100 nm) with a homogeneous distribution of particle diameter and lower polydispersity index. However, OAG presented a heterogeneous particle size and a high polydispersity index. Thus, these results confirmed those observed by SEM.

Lemos et al. [46] obtained microparticles with heterogeneous size distributions (132 to 490 μm), but he emphasized that the result obtained was within the expected range for particles obtained by coacervation. The researchers noted that the increased agitation speed led to a decrease in the size of the particles by promoting an increase in the system’s turbulence, contributing to the formation of smaller coacervates and, with this, smaller particles. In addition, studies have associated the influence of the concentration and type of surfactant on the homogeneous distribution of particle diameters in the incorporation efficiency of the core and, consequently, the physicochemical stability of the particles based on vegetable oils [50, 51]. Thus, it is believed that for the OAG formulation, it is necessary to employ rotation of the ultradisperser and surfactant concentration above those used in the present study, to obtain particles with smaller particle diameter and homogeneous size distribution.

Moser et al. [52] evaluated the encapsulation of buriti oil by atomization using a protein and carbohydrate complex containing chickpea protein and high-methoxyl pectin. The researchers also observed particles with a wide diameter range (0.4–300 μm), which was attributed to an early agglomeration process that promoted the formation of irreversible link bridges leading to the production of larger particles.

#### Zeta potential

This technique evaluates the charges present on the particles’ surface, which are influenced by changes in the interface with the dispersing medium due to the functional groups dissociating present or the ionic species adsorption present in the dispersing medium [53]. The zeta potential results obtained for OPG and OAG were 6.85 (0.81) mV and 10.34 (1.79) mV, respectively. The results indicated the presence of positive charges on the surface of the particles, showing the influence of pH on the isoelectric point of porcine gelatin since it has a positive charge at pH below 7.0 [54]. Since the alginate is anionic [18, 48], the particles obtained using the combination of encapsulating agents presented a predominance of gelatin on the surface.

The measurement values are also able to indicate the stability of the particles, thus influencing the definition of the applicability of encapsulates. According to Bhattacharjee (2016), particles are highly stable when zeta potential values are average ± 30 mV, moderately unstable by ±20–30 mV, and highly unstable when on average ± 0–10 mV. Thus, the particles obtained can be classified as highly unstable under neutral pH conditions evaluated in the study.

Wang et al. [55] promoted tuna oil encapsulation by the coacervation technique using gelatin and sodium hexametaphosphate and also obtained a similar average potential zeta value at pH values below 7.0. They observed the predominance of gelatin on the surface of the particles at a pH range of 4.0–7.0 due to the positively charged amine clusters (−NH^+ 3^) at the isoelectric point.

#### Fourier transform infrared spectroscopy (FTIR)

**Fig. 4:**
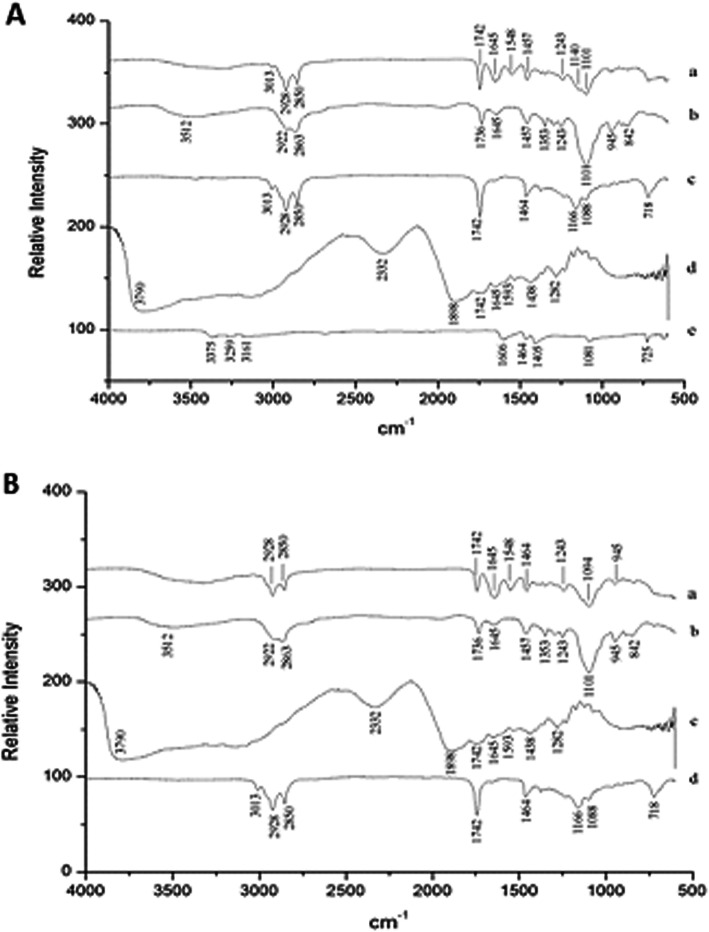
FTIR spectra of the formulations obtained by the O/W emulsification technique. (A) OAG: buriti oil, sodium alginate, and porcine gelatin: a. OAG, b. Tween 20, c. buriti oil, d. porcine gelatin, e. sodium alginate; (B) OPG: buriti oil, and porcine gelatin: a. OPG, b: Tween 20, c. porcine gelatin, d. buriti oil

In the buriti oil spectra (Fig. 4a and b), hydrocarbon groups were detected by the vibrational bands in the range of 2928–2850 cm^− 1^ (C-H bond), as well as bands in the region of 3013 cm^− 1^ and 1464 cm^− 1^, characterizing the –OH and –CH_3_ groups, respectively, demonstrating the presence of carotenoids in buriti oil as expected [4]. Besides, the vibration detected in the 1742 cm^− 1^ region indicates double bonds (C = C; C = O) in buriti oil, which characterizes the presence of unsaturated fatty acids.

The Tween 20 spectra (Fig. 4a and b) showed intense vibrations in the region of 3512 cm^− 1^, which characterizes the presence of –OH bonding; 2922 cm^− 1^ and 2863 cm^− 1^ for stretching of asymmetric and symmetrical methylene vibrations; 17,336 cm^− 1^, showing vibration of the carbonyl group; and 1101 cm^− 1^ for vibration stretching –CH_2_-O-CH_2_-, according to Silverstein & Webter [56].

In the porcine gelatin spectra (Fig. 4a and b), vibration at 1645 cm^− 1^ was observed, reflecting the presence of the C = O bond (amide I), which according to Silverstein & Webster [56], is close to the 1650 cm^− 1^ region. Another vibration is observed at 1593 cm^− 1^, indicating acyclic secondary amide as a function of N-H bond flexion, according to Guerrero et al. [57].

Sodium alginate (Fig. 4a) showed vibrations in the region of 1597–1413 cm^− 1^, indicating the presence of the carbonyl group (C = O), and similar results were also detected by Lawrie et al. [58] and Lemos et al. [46]. Besides, a vibration was observed in the range 3360–3156 cm^− 1^ that characterizes the presence of hydroxyl groups (O-H), which was also detected by Lee et al. [48].

When observing the OAG spectra (Fig. 4a), there was less attenuation in the bands for buriti oil (2928, 2850, 1742 cm^− 1^). However, attenuation of Tween 20 (1645 and 1101 cm^− 1^) bands and 1645 cm^− 1^ vibration stretching was also observed in porcine gelatin and Tween 20, and the formation of new bands (1548 cm^− 1^ and 1140 cm^− 1^) was identified, which may suggest the presence of chemical interactions between the materials used in the study.

The OPG spectra (Fig. 4b) showed that there was an interaction between buriti oil, porcine gelatin and Tween 20 surfactant by attenuation of the bands detected in Tween 20 (1097 cm^− 1^) and buriti oil (3013, 2928, 2850 and 1742 cm^− 1^), indicating both the presence and protection of the oil in the particles. Besides, new bands were formed in the region of 1548–1094 cm^− 1^, indicating hydrophobic interactions between nonpolar amino acids present in the porcine gelatin molecule and the carbonic chain of buriti oil.

Thus, in the present study, OPG presented more significant chemical interactions in its constituents than OAG, implicating the smaller particle diameter observed by laser diffraction. According to Karaca et al. [59], the physicochemical characteristics of proteins, such as solubility, molecular size, surface hydrophobicity, and molecular flexibility, directly influence their emulsifying properties. Therefore, the chemical interactions between proteins and lipids affect encapsulation.

#### X-ray diffraction (XRD)

**Fig. 5:**
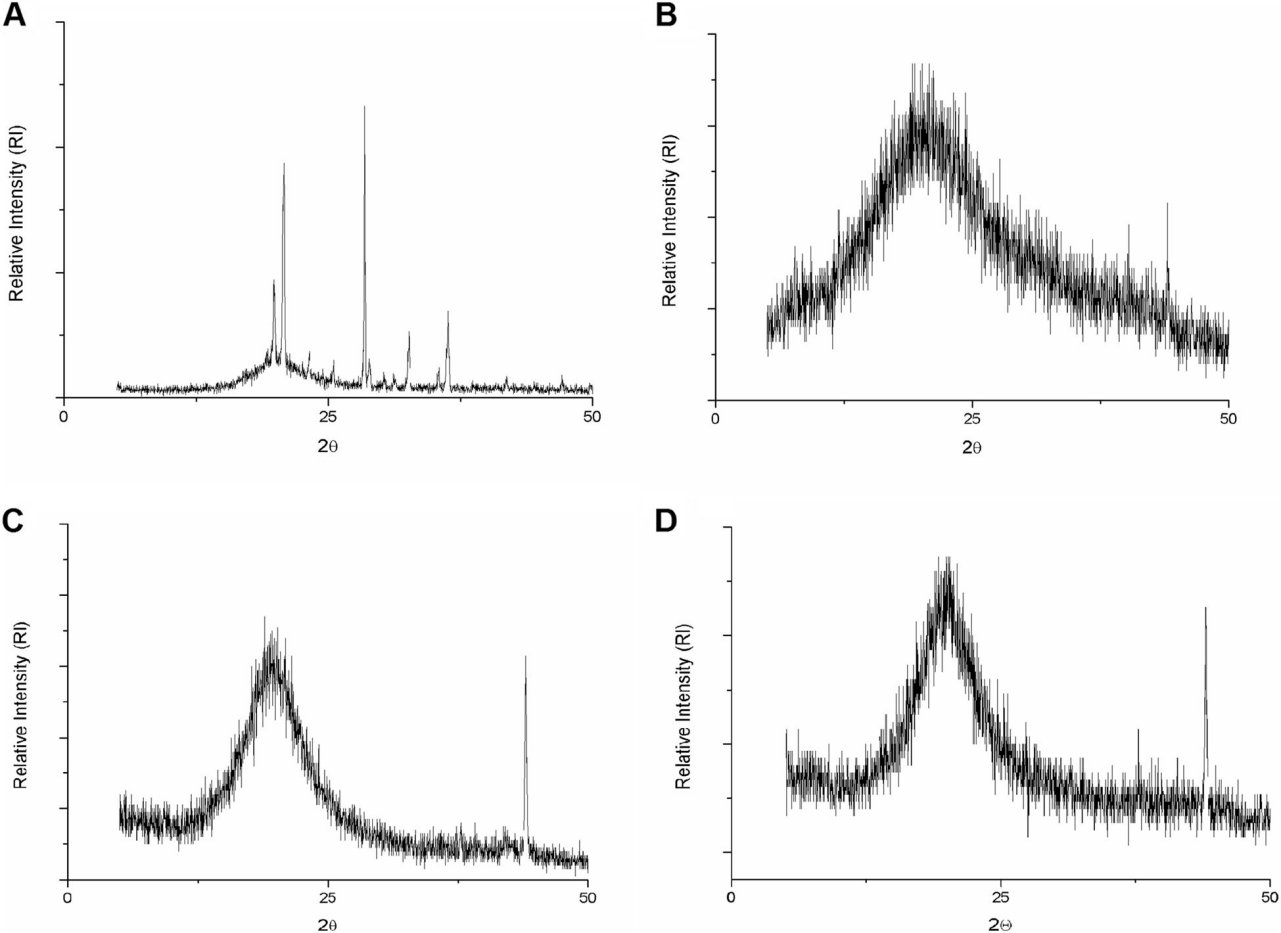
X-ray diffractograms of the powder particles obtained by the O/W emulsification technique. (A) Sodium alginate; (B) Porcine gelatin; (C) OAG: buriti oil, sodium alginate, and porcine gelatin; (D) OPG: buriti oil, and porcine gelatin

The diffractograms obtained for sodium alginate (Fig. 5a) and porcine gelatin (Fig. 5b) presented semicrystalline structures, with noises that characterize amorphous behavior and well-defined peaks, characterize crystalline regions, more predominant in alginate. Oliveira et al. [60] observed the same behavior for sodium alginate in a study involving the encapsulation of essential oil of rosemary. Alginate crystallinity is related to its molecular composition containing minerals such as sodium and calcium [61].

Detection of well-defined peaks in the gelatin diffractogram may be related to the structure of the triple collagen helix [62], which can also be observed in the diffractogram of OAG (Fig. 5c) and OPG (Fig. 5d) nanoformulations. Therefore, the diffractograms obtained for the formulations suggest the interaction between the buriti oil, encapsulating agents, and Tween 20, considering the peak displacements and attenuation in the intensity of the crystallinity signals observed.

### Buriti oil in particles (%)

The buriti oil (%) present in OPG and OAG were 86.80 (1.31) % and 71.91 (1.12) %, respectively. The results characterized a higher presence of buriti oil in particles containing gelatin than its combination with alginate (*p* < 0.05). Thus, the present study showed the excellent interaction between gelatin, buriti oil, and Tween 20, as observed in the FTIR characterization, which promoted more significant oil retention in OPG. This result can be explained because the proteins have physicochemical properties that favor the formation and stabilization of emulsions [59] compared to carbohydrates, thus allowing higher oil retention in the particles.

Although a low concentration of alginate was used in the OAG formulation compared to gelatin, the lower percentage of buriti oil observed in the particles may have been influenced by the space occupied by alginate, as pointed out by Soliman et al. [43].

### Water dispersion assay

Vegetable oils present the insolubility in water as a limitation. Nanoencapsulation may be a promising alternative for the application of vegetable oils to food matrices [63].

Nanoparticles have attracted attention to the development of new biopolymer complexes to improve solubility [64]. The particle size can directly influence solubility due to the larger contact surface for interactions between system constituents and the external environment. Thus, nanoparticles provide new functionality to the final product [47, 65].

**Fig. 6:**
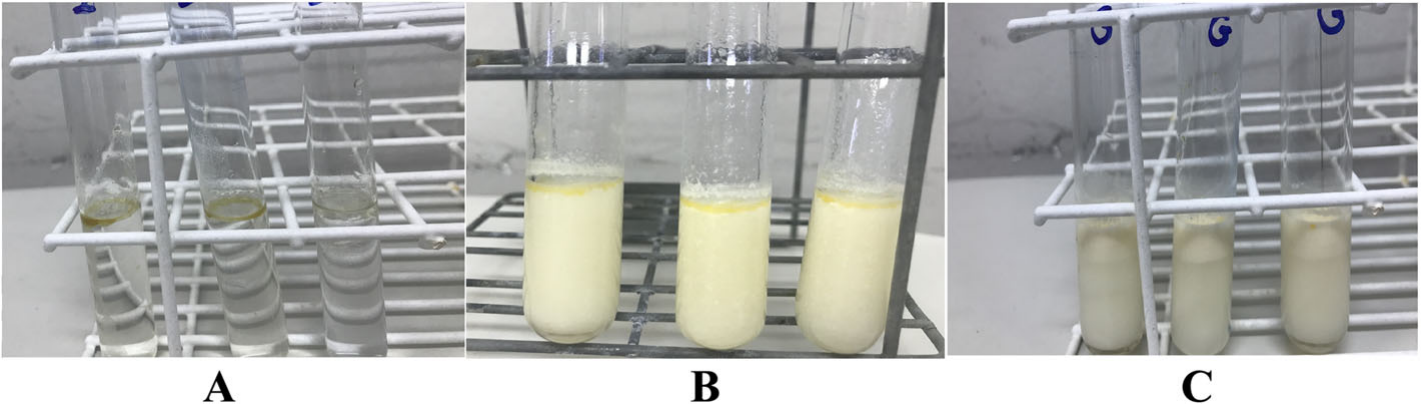
Water dispersion assay results of crude and encapsulated buriti oil obtained by the O/W emulsification technique. **a** Crude buriti oil; (**b**) OAG: buriti oil, sodium alginate, and porcine gelatin; and (**c**) OPG: buriti oil, and porcine gelatin

Crude buriti oil (Fig. 6a) had the lowest solubility percentage [3.91% (0.39)]. The results obtained for the encapsulates and crude buriti oil indicated that OPG (Fig. 6c) had higher dispersion in water [85.62% (7.82)] (p < 0.05) compared to OAG (Fig. 6b) [50.19% (7.24)]. Thus, these results showed that nanoencapsulation in gelatin and its combination with alginate enabled the water dispersibility of buriti oil by 77 and 45%, respectively. It is important to note that the amount of crude buriti oil present in formulations (OPG and OAG) was higher compared to crude buriti oil non encapsulated (20 mg). Two hundred milligram of OPG and OAG contained 75 mg and 93 mg of buriti oil, respectively.

Therefore, the chemical interaction between hydrophobic groups of the porcine gelatin and the oil chain, under the action of the Tween 20, provided a particle size reduction at the nanometer scale as observed in the characterization results, which directly contributed to the buriti oil dispersion in water. About the group of polysorbates, Tween 20 is the surfactant with the highest hydrophilic-lipophilic balance (HLB), approximately 16.7, to promote the stabilization of the interface between oil and water, allowing smaller particle sizes to be obtained [66].

### Antimicrobial activity

This evaluation was performed only in the crude buriti oil and OPG because only OPG presented good results, especially in the water dispersion assay. The crude oil (Table 3) showed low inhibition of bacterial growth of the strains analyzed compared to the OPG, indicating an increase of antimicrobial activity due to nanoencapsulation. The emulsification technique increased the antimicrobial activity of buriti oil by 59, 62, and 43% against *Pseudomonas aeruginosa*, *Klebsiella pneumonia,* and *Staphylococcus aureus,* respectively. Porcine gelatin was also used as a control at the same concentration present in OPG and did not inhibit the growth of the tested microorganisms. Leão et al. [7] obtained similar results for the antimicrobial activity of nanoemulsions based on interesterified and non-interesterified buriti oil in medium containing gram-negative bacteria (*E. coli*), achieving approximately 61% growth inhibition.

**Table 3:** Percentage of microbial growth inhibition for the microorganisms *Pseudomonas aeruginosa, Klebsiella pneumonia,* and *Staphylococcus aureus* by crude and nanoencapsulated (OPG) buriti oil (*Mauritia flexuosa*) at a concentration of 5 mg.ml^−1^

	Microbial growth inhibition (%)	
Microorganism	Crude buriti oil Mean (SD) %	OPG Mean (SD) %
*Pseudomonas aeroginosa*	16.43 (2.8)^a^	62.47 (3.00)^b^
*Klebsiella pneumonia*	22.51 (2.69)^a^	69.26 (1.79)^b^
*Staphylococcus aureus*	42.23 (1.30)^a^	66.94 (4.37)^b^

Mean and standard deviation (SD), *n* = 3

The different lowercase letters (a and b) in the same row indicate a statistical difference (*p* < 0.05), according to Student’s t-test

Particle size is an essential factor associated with antimicrobial activity [7, 64], which may have ensured that OPG, which presented the smallest particle size, had more significant inhibition of bacterial growth. Besides, the potentiation of antimicrobial activity may also be related to the increase in OPG dispersibility in water due to the size on a nanometric scale that promotes an increase in the oil’s contact surface with water [67].

The products of plant origin show more intense antimicrobial activity on gram-positive bacteria growth than gram-negative bacteria [68]. The cell wall of gram-negative bacteria can act as a barrier against bioactive compounds present in plant extracts and oils [68]. However, buriti oil has already been evaluated in different bacterial strains, showing more pronounced activity against *Bacillus subtilis, Klebsiella pneumoniae,* and *Staphylococcus aureus*. Therefore, it shows antimicrobial activity against both gram-negative and gram-positive bacteria [69]. Burh [70] associated the predominance of this antimicrobial action with the more significant interaction between the phytochemical constituents present in oils and the bacterial cell wall.

It should be noted that the present study obtained inhibition results above 50%, presenting itself above those obtained for non-encapsulated buriti oil, *Pseudomonas aeruginosa* and *Klebsiella pneumonia*, which are gram-negative microorganisms, using the OPG nanoformulation. Therefore, it is possible to observe the potential of nanoparticles containing buriti oil and porcine gelatin obtained by the O/W emulsification technique to be used in industrialized foods with aqueous matrices to enrich them with bioactive substances beneficial to human health. In addition to the potential for use in biodegradable food films to control the growth of microorganisms. Therefore, nanotechnology presents itself as a promising tool capable of revolutionizing the food industry, helping to solve various technological barriers that hinder the use of lipophilic ingredients with bioactive potential in industrialized foods.

## Conclusions

Based on these results, the encapsulation of buriti oil by the O/W emulsification technique, using porcine gelatin as an encapsulating agent and Tween 20 as a surfactant, was effective for the production of nanoparticles with a homogeneous size distribution, enabling the solubility of the oil in water and increasing its antimicrobial activity. These results enhance the potential application of buriti oil as an ingredient in industrialized foods.

## Data Availability

All data generated or analyzed during this study are included in this published article.
